# Scattered image artifacts from cone beam computed tomography and its clinical potential in bone mineral density estimation

**DOI:** 10.1186/s40064-016-3032-5

**Published:** 2016-08-18

**Authors:** Hoon Ko, Kwanmoon Jeong, Chang-Hoon Lee, Hong Young Jun, Changwon Jeong, Myeung Su Lee, Yunyoung Nam, Kwon-Ha Yoon, Jinseok Lee

**Affiliations:** 1 Department of Biomedical Engineering, Wonkwang University College of Medicine, Iksan Daero 460, Iksan, Jeonbuk 54538 Republic of Korea; 2 Imaging Science Based Lung and Bone Disease Research Center, Wonkwang University, 460 Iksandeaero, Iksan, Jeonbuk 54538 Republic of Korea; 3 Department of Rheumatology Internal Medicine, Wonkwang University School of Medicine, Iksan, Jeonbuk 54538 Republic of Korea; 4 Department of Computer Science, Soonchunhyang University, Cheonan, Chungnam 31538 Republic of Korea; 5 Department of Radiology, Wonkwang University School of Medicine, Iksan Daero 460, Iksan, Jeonbuk 54538 Republic of Korea

**Keywords:** Scattered artifact image, Bone mineral density, Osteoporosis, Cone-beam computed tomography

## Abstract

**Background:**

Image artifacts affect the quality of medical images and may obscure anatomic structure and pathology. Numerous methods for suppression and correction of scattered image artifacts have been suggested in the past three decades. In this paper, we assessed the feasibility of use of information on scattered artifacts for estimation of bone mineral density (BMD) without dual-energy X-ray absorptiometry (DXA) or quantitative computed tomographic imaging (QCT).

**Methods:**

To investigate the relationship between scattered image artifacts and BMD, we first used a forearm phantom and cone-beam computed tomography. In the phantom, we considered two regions of interest—bone-equivalent solid material containing 50 mg HA per cm^−3^ and water—to represent low- and high-density trabecular bone, respectively. We compared the scattered image artifacts in the high-density material with those in the low-density material. The technique was then applied to osteoporosis patients and healthy subjects to assess its feasibility for BMD estimation.

**Results:**

The high-density material produced a greater number of scattered image artifacts than the low-density material. Moreover, the radius and ulna of healthy subjects produced a greater number of scattered image artifacts than those from osteoporosis patients.

**Conclusions:**

Although other parameters, such as bone thickness and X-ray incidence, should be considered, our technique facilitated BMD estimation directly without DXA or QCT. We believe that BMD estimation based on assessment of scattered image artifacts may benefit the prevention, early treatment and management of osteoporosis.

## Background

Medical image artifacts are commonly encountered in medical imaging by X-ray computed tomography (CT), ultrasonography and magnetic resonance imaging (MRI). Such artifacts affect image quality and may obscure anatomic structure and pathology (Anas et al. 2011; Boas and Fleischmann 2012). To increase image quality, several types of artifact—such as beam hardening, scattering, motion, helical, ring and metal artifacts—have been the subjects of investigation (Glover and Pelc 1981; Huang et al. 2015; Mřnch et al. 2009; Yazdi and Beaulieu 2008; Zhang et al. 2007).

This study focused on scattered image artifacts from cone-beam computed tomography (CBCT). Scattered image artifacts produce dark streaks between two high-attenuation objects (such as metal, bone, iodinated contrast and barium) with surrounding bright streaks (Huang et al. 2015; Schulze et al. 2014; Zhen et al. 2012). Numerous methods for suppression and correction of scattered image artifacts have been suggested in the past three decades (Ogawa et al. 1991; Ohnesorge et al. 1999; Siewerdsen et al. 2006; Sun et al. 2011). However, the complete correction of scattered image artifacts in CT images remains a challenge.

The focus of this work was not on reducing the number of scattered artifacts, but on making use of information on scattered artifacts to facilitate bone mineral density (BMD) estimation without dual-energy X-ray absorptiometry (DXA) or quantitative computed tomographic imaging (QCT). Indeed, BMD estimation based on scattered image artifacts was first attempted in the 1980s (Huddleston and Weaver 1983). However, CBCT was not available in the 1980s; it was introduced in the US in 2001 (Hatcher 2010).

In this paper, the effect of substance density on scattered image artifacts using CBCT was evaluated using a forearm phantom containing cortical walls and trabecular bones, the densities of which differed according to their hydroxyapatite (HA) content (200, 100, 50 or 0 mg cm^−3^). The abundance of scattered image artifacts was compared between low- and high-density regions of interest. The scattered image artifacts technique was then applied to osteoporosis patients and healthy subjects.

## Methods

### Scattered Radiation

The number of scattered photons based on Compton interactions is proportional to the electron density of a substance. Given a sphere with radius *r*, let the number of scattered photons from the center of a sphere be denoted by $dI_{s} \left( r \right),$ which can be formulated as follows (Huddleston and Weaver 1983):

1 $$dI_{s} \left( r \right) \sim \frac{I\left( r \right)}{A\left( r \right)} \cdot \exp \left( { - \mu \left( E \right) \cdot r} \right) \cdot \rho \cdot \theta \left( r \right),$$

where $I\left( r \right)$ is the number of photons scattered to the detector per unit volume, $A\left( r \right)$ is the area of the beam, $\mu \left( E \right)$ is the linear attenuation coefficient of the sphere substance at the incident photon energy *E*, $\rho$ is the electron density of the sphere substance and $\theta \left( r \right)$ is the solid angle originating at the center. Then, by integrating $dI_{s} \left( r \right)$ through the entire sphere, the total number of scattered photons can be calculated as:

2 $$I_{s} \sim \exp \left( { - \mu \left( E \right) \cdot r} \right) \cdot \rho \cdot S,$$

where *S* is the average scatter intensity per electron. Thus, the number of scattered artifacts is proportional to the attenuation coefficient, thickness and density of a substance. Therefore, given an identical X-ray incidence and substance thickness, the density can be estimated using the abundance of scattered image artifacts.

### Forearm phantom

**Fig. 1:**
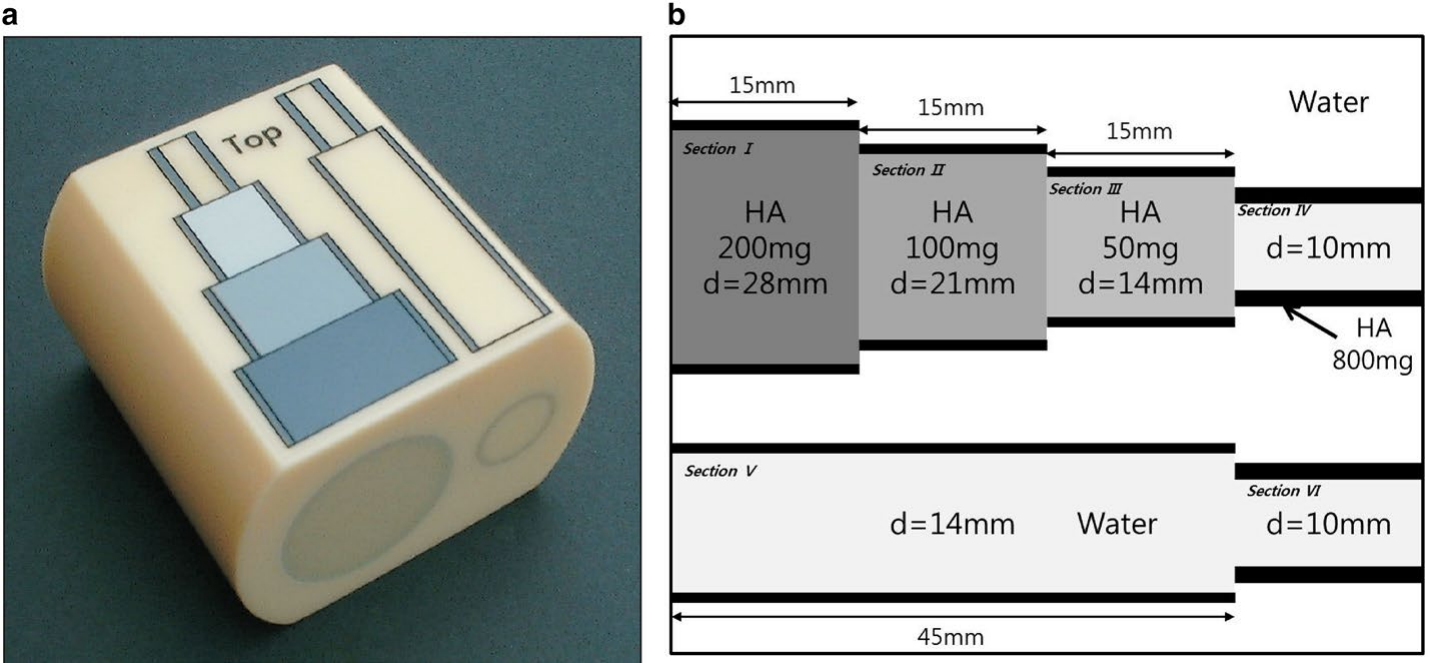
Medicine European forearm phantom (QRM-EFP); **a** QRM-EFP appearance and **b** its inner structure

To investigate the relationship between the abundance of scattered image artifacts and BMD, a forearm phantom consisting of materials of various densities was used. The Medicine European Forearm Phantom (QRM-EFP) comprises water- and bone-equivalent solid materials and is used to test peripheral bone densitometry systems (Ruegsegger and Kalender 1993). The QRM-EFP is manufactured from a resin-based water-equivalent material and contains cortical walls and trabecular bones of various densities (200, 100 and 50 mg HA cm^−3^, representing high-, medium- and low-density bone, respectively) (Fig. 1). The cortical walls are of thickness 1.2 and 2.5 mm and density 800 mg HA cm^−3^ (Augat et al. 1998; Ruegsegger and Kalender 1993).

### Acquisition of scattered image artifacts using peripheral CBCT

**Fig. 2:**
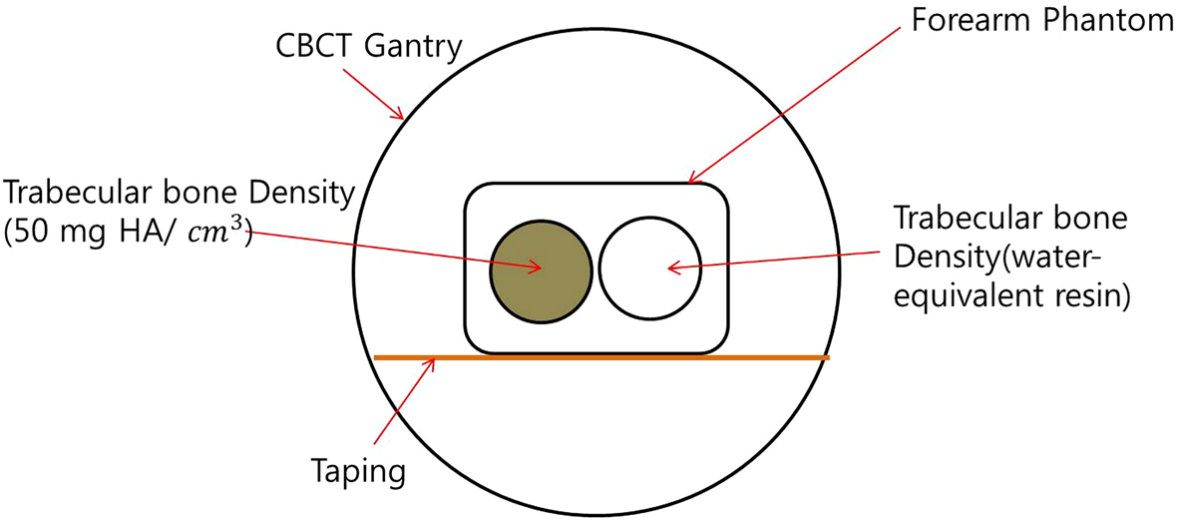
Cross-sectional view of QRM-EFP scanning from CBCT

We obtained phantom images of the QRM-EFP forearm using peripheral CBCT (PHION, Nano Focus Ray, Jeonju, Korea). The scanning parameters were 130 kVp, 10 mA, scanning times of 15–20 ms and a 1024 × 1024 image matrix. The total number of image slices was 462, each of 0.2162 mm thickness. To avoid generation of artifacts due to reflection from the underlying table, we suspended the phantom in the air (Fig. 2).

**Fig. 3:**
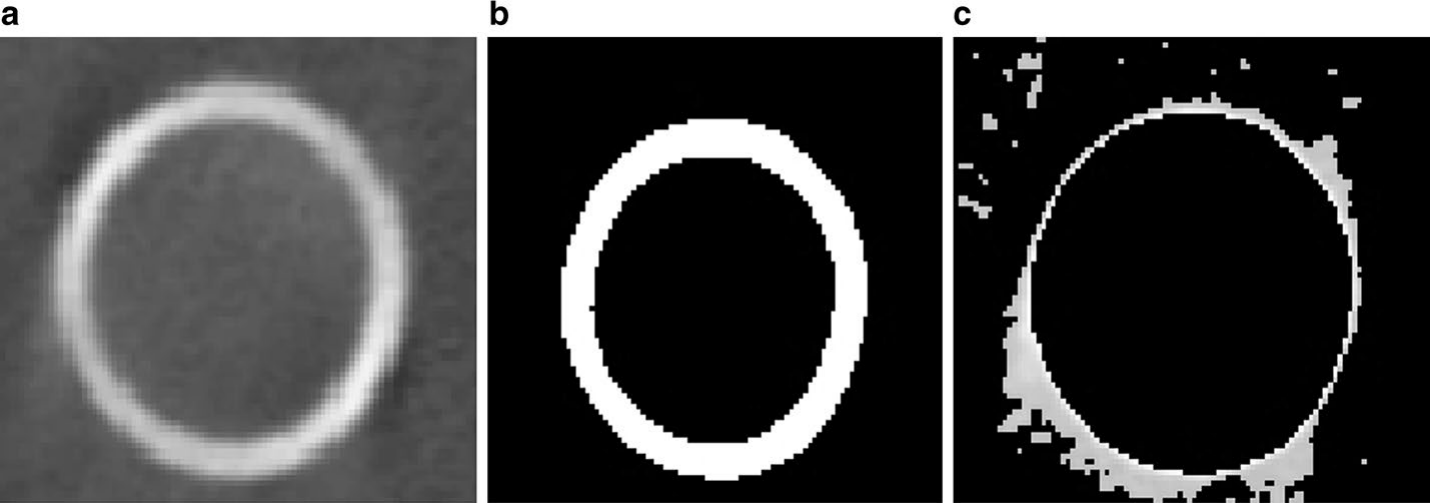
Scattered image artifacts acquisition **a** original QRM-EFP images obtained from CBCT, **b** separation of the cortical wall, and **c** remained scattered image artifacts

Figure 3a shows a QRM-EFP image obtained by peripheral CBCT. Using this image, the cortical wall contour was extracted as shown in Fig. 3b. The cortical bone compartment was segmented by an auto-contouring process, which generates a periosteal contour that delineates mineralized bone and extra-osseal soft tissue, and an endosteal contour that delineates the endocortical boundary from the cancellous compartment (Ma and Manjunath 2000; Shah et al. 2006). To extract the periosteal surface, thresholding using a value of 900 Hounsfield units (HU) was applied to the CT image to obtain the contour of cortical bone. Clinically, the threshold HU value can differ among anatomical locations (De Oliveira et al. 2008; Hu et al. 2000; Richter et al. 2008). The endosteal surface was then extracted by masking the periosteal surface with a bone mask from the original image, and the periosteal contour was removed to leave only the pixels representing the marrow (Defreitas 1999). Subsequently, the area inside the periosteal surface was subtracted so that only the scattered image artifacts remained, as shown in Fig. 3.

**Fig. 4:**
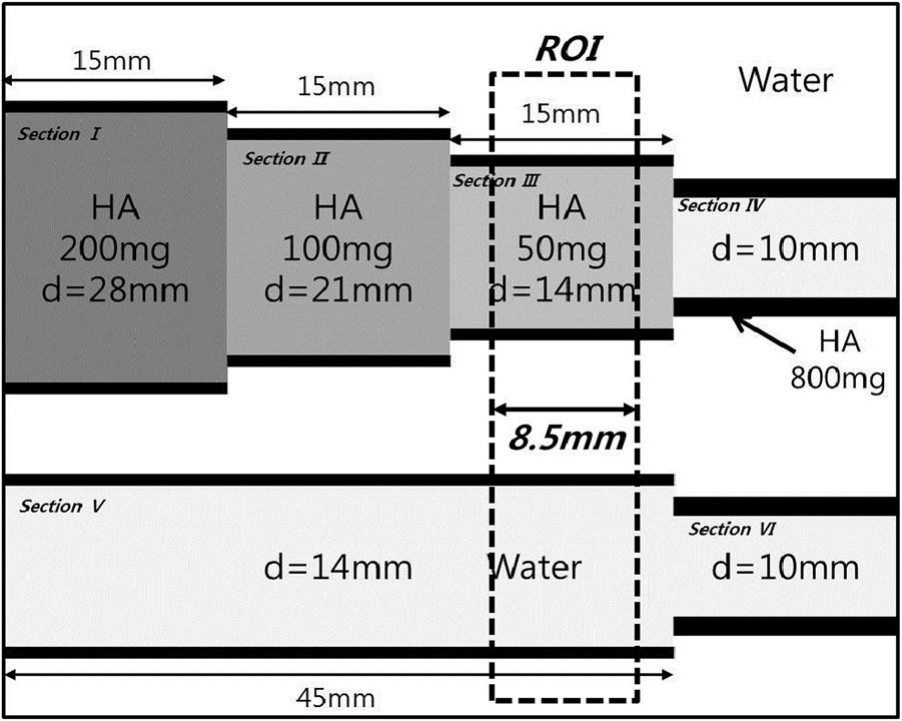
Two regions of interest (ROIs) representing low and high density of a trabecular bone

Among the extracted scattered image artifacts from 462 slice images, we considered 40 slice images corresponding to the two regions of interest (“Results”, “Discussion” sections) (Fig. 4). Each ROI corresponded to bone-equivalent solid material containing 50 mg HA per cm^−3^ and water, representing low- and high-density trabecular bone, respectively. To evaluate the effect of density on scattered artifacts, threshold HU values of 500–900 in increments of 20 HU were applied, and the artifact pixels in low- and high-density materials were enumerated. The number of artifact pixels in the high-density material was subtracted from that in the low-density material.

## Results

### Phantom study

**Fig. 5:**
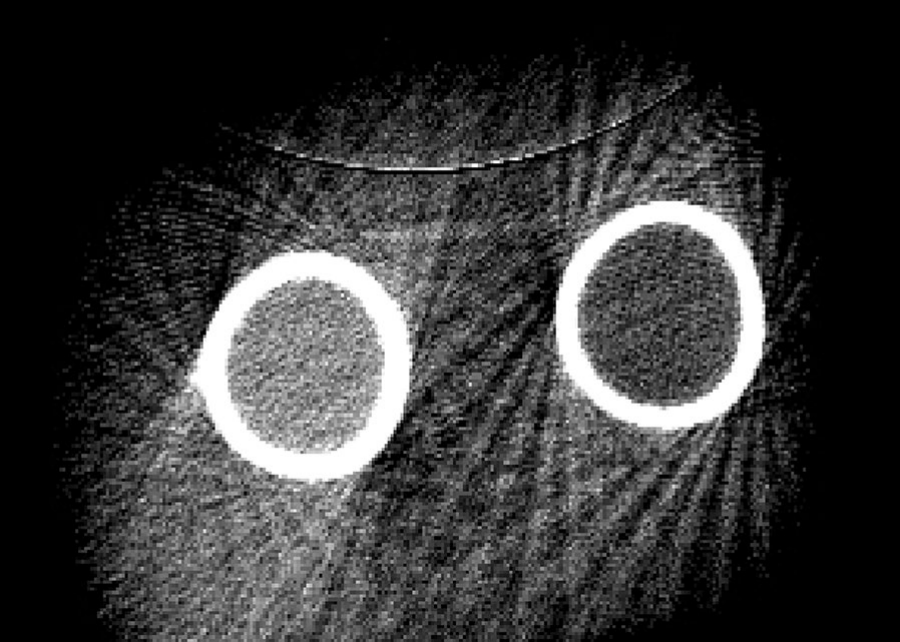
The scanned QRM-EFP images obtained from peripheral CBCT with 130 kvp, 10 mA and 20 ms exposure time

**Fig. 6:**
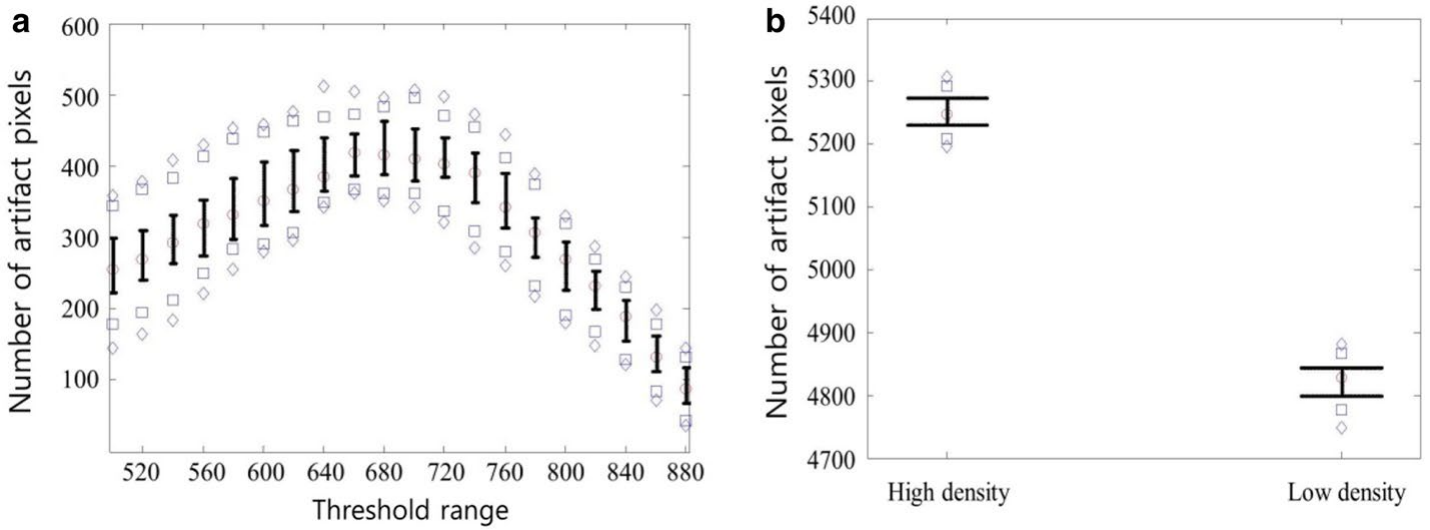
**a** The subtracted values (the number of artifacts from the high density material minus the number of artifacts from low density material) according to the threshold values, and **b** the number of artifact pixels from low- and high-density material; The *diamonds* above and below represent the 5th and the 95th percentiles of each group, and the *squares* above and below represent the 90th and the 10th percentiles. *Whiskers* above and below represent the 75th and the 25th percentiles, respectively. The *circle* is the median value

Figure 5 shows a CBCT image of the QRM-EFP obtained with 130 kVp, 10 mA and a 20 ms X-ray exposure time. The left and right circles represent high- and low-density material, respectively. The results suggest that the high-density material produces a greater number of scattered image artifacts than does the low-density material. Scattered image artifacts were further investigated by varying the threshold HU values from 500 to 900 with an increment of 20 HU and enumerating the scattered artifact pixels from low- and high-density materials. The number of scattered image artifacts from the high-density material was subtracted from that of the low-density material (Fig. 6a). The number of scattered image artifacts was highest using a threshold value of 660, which yielded 5169 ± 37 scattered image artifacts from the low-density material and 5590 ± 32 from the high-density material (Fig. 6b).

### Clinical results from pilot study

**Fig. 7:**
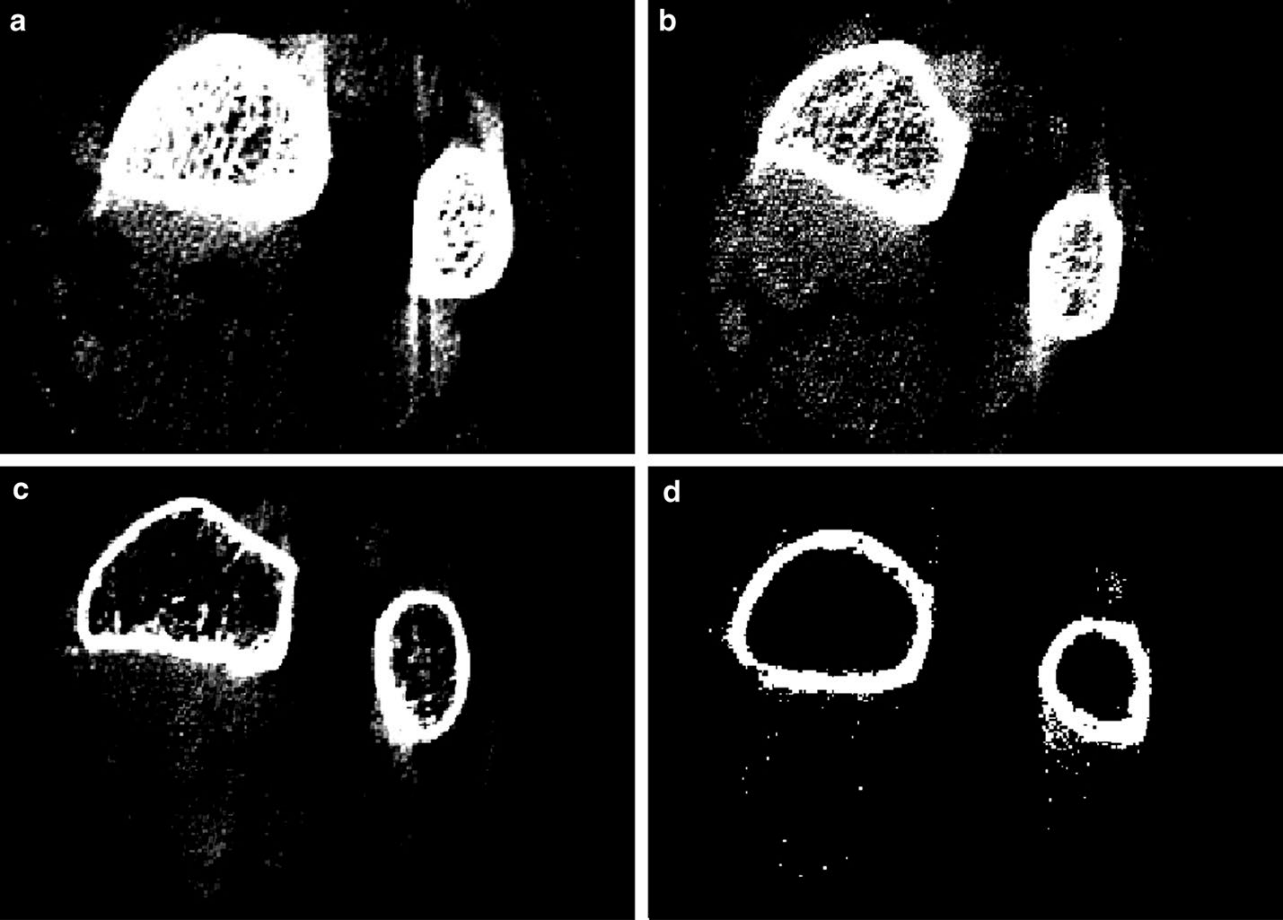
Scanned forearm CBCT images; **a**, **b** From two healthy subjects, **c**, **d** From osteoporosis patients

**Fig. 8:**
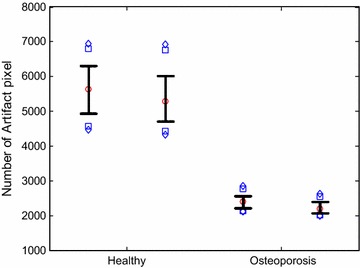
Comparison of the number of scattered artifact pixels from two healthy subjects and two osteoporosis patients

Using the procedure outlined above, a pilot study involving two osteoporosis patients and two healthy subjects using CBCT was performed. Written informed consent was obtained from the patient for publication of this case report and any accompanying images. The scanning parameters were 130 kVp, 8 mA, scanning time of 7.5 s, and image matrixes of 1024 × 1024 pixels with a slice thickness of 0.2162 mm. The scanning region extended roughly from the carpal bone to the elbow. Scattered image artifacts were enumerated using the optimal threshold value determined by two radiologists. The measurement and image analysis were performed by two radiologists at Wonkwang University Hospital (WKUH). The WKUH Institutional Review Board (IRB) approved the measurement and analysis protocol. Figure 7a–d shows the scanned CBCT images of the forearm of the two healthy subjects and the two osteoporosis patients, respectively. The radius and ulna of healthy subjects produced a greater number of scattered image artifacts than did those of the osteoporosis patients. The number of scattered artifact pixels was 2332 ± 229 from the two osteoporosis patients and 5572 ± 831 from the two healthy subjects (Fig. 8).

## Discussion

Using the QRM-EFP, we scanned and acquired BMD values for the ROI (50 g/cm^3^) from DXA and QCT, and the values were 0.048 g/cm^3^ and 0.052 g/cm^3^, respectively, which were within the tolerance range in the phantom specification. In this study, we presented feasibility study that high-density material produced a greater number of scattered image artifacts than the low-density material. In addition, we further presented feasibility for the screening of osteoporosis. To quantify BMD value from the amount and/or intensity of artifacts, more sophisticated material density intervals in phantoms or clinical data with optimized parameters of scanning time, image matrix, voltage and current need be further investigated in the future work.

In our study, we used peripheral CBCT, which was recently introduced two decades ago and currently and widely used for clinical diagnosis. It provides two-dimensional and three-dimensional images for the radiographed area with a relatively low cost radiation dose as compared to conventional CT. Due to the low cost and low radiation dose, it became popular for peripheral site scanning, which can simply scan the extremity in a convenient way (ex. in a sitting or standing posture) rather than in a supine position using QCT and DXA. Regarding the radiation dose, DXA is with less radiation dose than CBCT. For an adult forearm, the effective radiation dose from CBCT is approximately 0.04 mSv, which is less than that from multi-detector CT (MDCT) with 0.13 mSv, but higher than that from DXA with 0.03 µSv (Huang et al. 2015; Thomas et al. 2006). However, in our study, only one slice image with thickness of 0.2162 mm out of 462 slice images is enough to assess the BMD value. Then, assuming the CBCT detector is optimized with the corresponding thickness, the radiation dose can be further reduced up to 1/462 times, which is approximately equivalent to 0.3 µSv. Furthermore, the forearm CBCT images can be used for estimating BMD values of lumbar spine and hip bone with a linear regression (Jeong et al. 2016). Then, for lumbar spine and/or hip bone BMD, the effective radiation dose from CBCT is less than that from DXA, 2.2 µSv (Thomas et al. 2006).

One of the advantages in our approach is that only a forearm needs to be exposed via X-ray for BMD assessment of a lumbar spine and/or hip bones since the forearm CBCT images can be used for estimating BMD values of a lumbar spine and hip bones (Jeong et al. 2016). On the other hand, traditional approaches using DXA and QCT require X-ray exposure in the entire axial skeleton near a lumbar spine or a hip. Since the cancer occurrence by X-ray exposure is one of the main reasons, the exposure of the forearm is better than that of the axial skeleton. Another advantage in our approach is that the radiation dose can be further reduced since only the artifacts images are used for BMD estimation. Much research efforts on BMD estimation have been conducted based on the correlation with HU and the image quality improvement of the bone ROI. More specifically, in Islamian et al. (2016), the effect of intravenous contrast media was investigated on BMD measurement of lumbar spine vertebrae with CT densitometric data. In (Schreiber et al. 2011), the correlation relationship was studied on HU with BMD and compressive strength. In Pickhardt et al. (2011), the BMD assessment was studied on phantomless QCT and simple ROI attenuation measurements of the lumbar spine. In (Islamian et al. 2016), BMD of lumbar spine vertebrae was investigated with CT densitometric data on HU from abdominal and lumbar spine CT examinations. In Li et al. (2013), the osteoporosis detection rates in postmenopausal were compared from BMD with QCT and DXA in the ROI, spine images. In Bauer and Link (2009; Buckens et al. 2015; Majumdar and Leslie 2013), the osteoporosis screening based on QCT and/or DXA was similarly investigated. On the other hand, we used the amount of scattered artifacts outside the ROI, which is available even with low radiation dose. Thus, more research on the low radiation dose needs to be investigated in the future work.

**Fig. 9:**
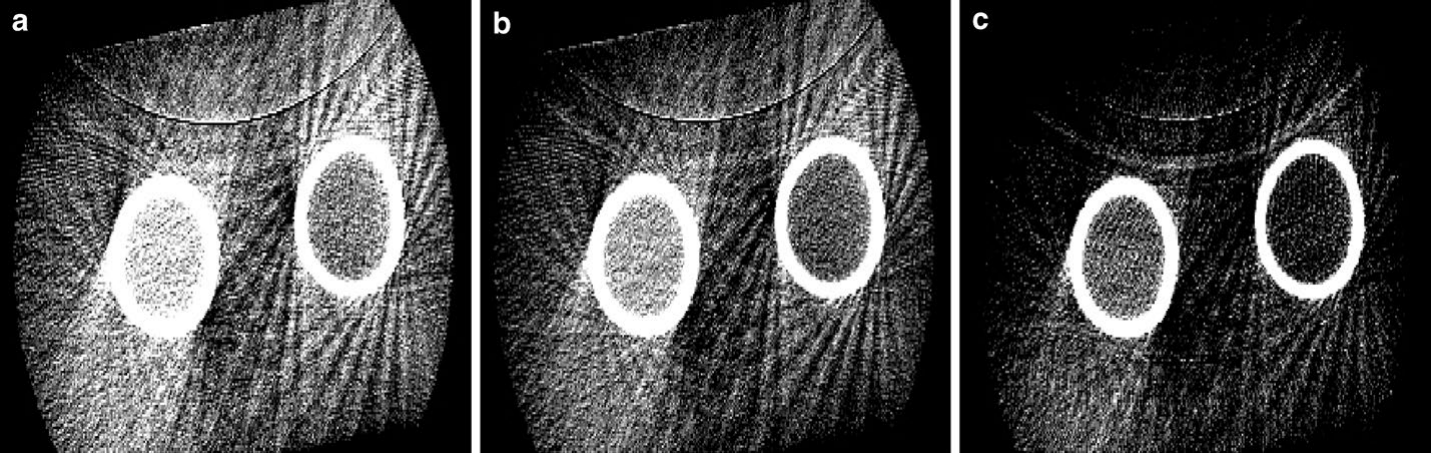
Effect of X-ray tube voltage on scattered image artifacts. **a** 100 kvp, 10 mA, 20 ms, **b** 110 kvp, 10 mA, 20 ms, and **c** 120 kvp, 10 mA, 20 ms

However, even though the scattered image artifacts using CBCT simply and conveniently provide the bone density information, the other parameters of attenuation coefficient and bone thickness are also proportional to the scattered artifact intensity. Figure 9 shows the scattered image artifacts according to x-ray tube voltages, where x-ray tube current and scanning time is fixed with 10 mA and 20 ms, respectively. Thus, an optimum x-ray incident as well as a bone thickness should be considered to be clinically available in the future. Furthermore, the pilot study needs to be rigorously further validated by considering more extensive subjects.

## Conclusion

We investigated the effect of substance density on the production of scattered image artifacts using CBCT. The high-density material in a forearm phantom produced more scattered image artifacts than did the low-density material. To validate this finding, we applied the technique to osteoporosis patients and healthy subjects as a pilot study, and found that the osteoporosis patients produced fewer scattered image artifacts than the healthy controls. Although other parameters, such as bone thickness and X-ray incidence, should be considered, our technique facilitates BMD estimation without DXA or QCT. Therefore, BMD estimation based on scattered image artifacts may benefit the prevention, early treatment and management of osteoporosis.
